# Editorial: Exploring cardiovascular and cerebrovascular diseases interaction with inflammation: biomarkers, drug targets, and personalized treatments through multi-omics data integration

**DOI:** 10.3389/fimmu.2025.1619519

**Published:** 2025-06-27

**Authors:** Qikai Tang, Chenfeng Ma, Yacheng Shi, Jian Song, Yong Cao, Wei Wu

**Affiliations:** ^1^ Department of Neurosurgery, First Affiliated Hospital of Nanjing Medical University, Nanjing, China; ^2^ Department of Neurology, The Affiliated Wuxi People’s Hospital of Nanjing Medical University, Wuxi, China; ^3^ Institute of Physiological Chemistry and Pathobiochemistry and Cells-in-Motion Interfaculty Centre (CIMIC), University of Münster, Münster, Germany; ^4^ Department of Neurosurgery, Beijing Tiantan Hospital, Capital Medical University, Beijing, China

**Keywords:** cardiovascular and cerebrovascular diseases, inflammation, multi-omics biomarkers, personalized treatment strategies, drug target discovery, immune modulation

Cardiovascular and cerebrovascular diseases (CVDs) remain the leading causes of mortality worldwide (1). An increasing body of evidence highlights that inflammation is not merely a bystander but a central driver of the initiation, progression, and complications of these diseases (2). Metabolic dysregulation and immune system dysfunction intricately intertwine, accelerating vascular injury and adverse clinical outcomes (3). Although significant advances have been made in understanding the metabolic aspects of CVDs, the inflammation-centric pathophysiological mechanisms are less comprehensively explored. This Research Topic aimed to bridge this gap by focusing on the integration of multi-omics technologies—genomics, transcriptomics, proteomics, metabolomics, and immunomics—to unravel new biomarkers, identify novel drug targets, and enable personalized treatment strategies.

The studies included in this Research Topic present a comprehensive view of the interplay between metabolism, inflammation, and vascular diseases, underscoring the potential of multi-omics integration in transforming cardiovascular and cerebrovascular medicine.

## Unveiling disease mechanisms through multi-omics integration

Several contributions in this Research Topic employed integrated omics approaches to decipher the molecular underpinnings of CVDs and their inflammatory components. Wang et al. discovered the ribosome biogenesis genes and subgroups in ischemic stroke. Rodríguez-González et al. revealed the relationship between the complement system and blood lipids in patients with rheumatoid arthritis. transcriptomic and proteomic profiling revealed key differentially expressed genes and proteins involved in immune cell regulation, oxidative stress, and endothelial dysfunction. These findings not only deepened our understanding of disease mechanisms but also highlighted the heterogeneity among patient subgroups, emphasizing the need for personalized diagnostic and therapeutic strategies.

A central theme emerging from this Research Topic is the discovery of novel biomarkers with potential clinical relevance. Shi et al. discovered the early and late corticospinal tract injuries and cytokine responses in patients with acute unilateral brainstem infarction. Using machine learning algorithms and high-dimensional omics datasets, researchers identified key genes, circulating proteins, and metabolites predictive of disease onset, severity, and therapeutic response. Li et al. evaluated the relationship between NETs and the inflammatory risk of large artery atherosclerotic stroke and its clinical predictive value.

Targeted interventions aimed at modulating these molecules offer new avenues for therapeutic development, particularly for patients who do not adequately respond to traditional risk factor management.

## Translating multi-omics discoveries into clinical applications

An important strength of the articles included is their focus on the translational aspects of omics discoveries. Several studies emphasized the development of clinically applicable diagnostic tools based on identified biomarkers, including multiplex assays and predictive models. Zhang et al. comprehensively explained the inflammatory left and right sides of cardiovascular and cerebrovascular diseases. Zhu et al. Systematically expounded Mitophagy-associated programmed neuronal death and neuroinflammation.

Moreover, efforts to stratify patients based on molecular profiles pave the way for personalized treatment approaches. For example, integrating genetic susceptibility, inflammatory biomarkers, and metabolic profiles enables the identification of high-risk individuals who may benefit from aggressive preventive interventions or targeted therapies. Wang et al. found that PDPN in astrocytes reduces hippocampal inflammation in T2DM mice. Liu et al. discovered the role of the IL-7R gene in the diagnosis of post-stroke depression.

In addition, clinical trials exploring anti-inflammatory and immunomodulatory agents were discussed, highlighting the critical need for evidence-based strategies to translate laboratory findings into effective clinical interventions. Zhao et al. revealed the protective effect of negative feedback regulation of the CD73-A2AR axis through the NF-κB pathway in cirrhotic cardiomyopathy.

## Challenges and future perspectives

Despite the promising advances highlighted in this Research Topic, several challenges remain. Integrating multi-omics data across platforms, sample types, and populations requires standardized methodologies and robust computational tools (4). Furthermore, the functional validation of candidate biomarkers and therapeutic targets remains crucial to ensure biological relevance (5).

Large-scale, multi-center studies are needed to validate findings across diverse patient populations and disease subtypes (6). Importantly, ethical considerations surrounding data privacy, particularly in the context of genomics and personalized medicine, must be addressed.

Moving forward, interdisciplinary collaboration between clinicians, bioinformaticians, and basic scientists will be essential to fully harness the power of multi-omics technologies in cardiovascular and cerebrovascular disease research.

## Conclusion

The Research Topic “Exploring Cardiovascular and Cerebrovascular Diseases Interaction with Inflammation: Biomarkers, Drug Targets, and Personalized Treatments through Multi-omics Data Integration” highlights the transformative potential of multi-omics approaches in understanding and managing CVDs. By elucidating the complex interplay between metabolic and inflammatory processes, identifying novel biomarkers and therapeutic targets, and paving the way for personalized interventions, these studies set the stage for a new era in cardiovascular and cerebrovascular medicine.

We sincerely thank all contributing authors, reviewers, and editorial staff for their invaluable efforts. We look forward to the continued advancement of this exciting field and the translation of these discoveries into improved outcomes for patients worldwide.
